# Supplementary material for: An aging-independent replicative lifespan in a symmetrically dividing eukaryote
Source: eLife. 2017 Jan 31;6:e20340. doi: 10.7554/eLife.20340 (PMC5332158; doi:10.7554/eLife.20340)
Supplement: Supplementary file 1. — It references four works not referenced in the main text: Wood and Nurse, 2013; Xhemalce and Kouzarides, 2010; Weisman et al., 2005; Nakazawa et al., 2008 DOI: http://dx.doi.org/10.7554/eLife.20340.024 [file elife-20340-supp1.docx]

**Supplemental Table 1. Comparison of cell metrics to literature**

| **Measurement** | **This Research** | **Literature** | **Ref** |
| --- | --- | --- | --- |
| Length at Birth (µm) | 8.0 ± 1.5 (*n = 13,905*) | 8.2 ± 0.52 (*n = 164*) 8.7 ± 0.9 (*n ≤ 900*) | (12)  (78) |
| Length at Division (µm) | 16.0 ± 2.2 (*n = 13,456*) | 14.4 ± 0.85 (*n = 164*) 14.7 ± 1.2 (*n ≤ 900*) | (12)  (78) |
| Cycle Time (m) | 122 ± 28 (*n = 13,456*) | 148 ± 16 (*n = 164*) 133 ± 16 (*n = 72*) | (12)  (15) |
| Rate Change Point | 0.36 ± 0.07 (*n = 50*) | 0.34 (*n = 5*5) | (12) |
| % Change of Rate at RCP | 0.22 ± 0.19 (*n = 33*) | 0.31 (*n = 55*) | (12) |

All cells were grown in the FYLM with YES media at 31°C. Rate Change Point (RCP) is the position of the new end takeoff rate change in a fractional measurement of cell cycle. “% Change of Rate at RCP” is the change in slope of a linear regression performed on smoothed data after the RCP with respect to the slope of a linear regression performed on smoothed data before the RCP. Measurements are presented as mean ± standard deviation. Cells for reference (12) in the table were grown on an agar substrate made with conditioned EMM3 at 35°C. Cells for reference (78) in the table were grown using YE4S medium in a microfluidic device at 32°C. Cells for reference (15) in the table were grown on an agar substrate made with YES (1% Yeast Extract, 3% glucose) at 30°C.

**Supplemental Table 2. Mean RLS and hazard rates for key strains**

| **strain** | **n** | **Mean RLS (95% CI)** | **Age-independent rate (gen^-1^)** | **Age-dependent rate (gen^-1^)** | **r^2^_adj_** |
| --- | --- | --- | --- | --- | --- |
| Wild-type (h- 972) | 440 | 39.2 (38.6-39.8) | 0.017 ± 0.001 | N.A. | 0.99 |
| *rqh1Δ* | 126 | 5.3 (5.0-5.7) | 0.130 ± 0.009 | N.A. | 0.95 |
| *rqh1Δ sir2*OE ^§^ | 96 | ~2^§^ | N.A. | N.A. | N.A. |
| *rqh1Δ* + rapamycin | 63 | 6.9 (6.2-7.2) | 0.10 ± 0.01 | N.A. | 0.96 |
| WT + rapamycin | 184 | 55.3 (53.1-57.6) | 0.013 ± 0.001 | N.A. | 0.96 |
| *sir2Δ* | 329 | 33.4 (32.6-34.3) | 0.021 ± 0.001 | N.A. | 0.98 |
| *sir2*OE | 301 | > 60 | 0.009 ± 0.001 | N.A. | 0.98 |
| WT (CBS2777) | 100 | 22.7 (21.9-23.6) | 0.031 ± 0.002 | N.A. | 0.98 |
| WT (NCYC132) | 226 | > 50 | 0.003 ± 0.001 | N.A. | 0.97 |
| WT (JB760) | 147 | >70 | 0.006 ± 0.001 | N.A. | 0.98 |
| S. cerevisiae | 458 | 25.6 (23.6-28.0) | 0.005 ± 0.001 | 0.106 ± 0.005 | 0.99 |

Survival curves were fit with either an exponential decay or a Gompertz function, yielding fit values and 95% confidence intervals for α and β. RLS was calculated using either Equations 6 or 7. “n” denotes the number of cells tracked in each experiment. “r^2^_adj_” is the r^2^ value of the fit adjusted for the number of coefficients. N.A.: Not any.

§ The extremely short lifetime of this strain precluded an accurate fit. We estimate the RLS as the number of generations required to cause death of 50% of the cells that were loaded into the multFYLM.

**Supplemental Table 3. Strains used in this study**

| **Identifier** | **Description** | **Alias** | **Mat.** | **Source** |
| --- | --- | --- | --- | --- |
| IF30 | wildtype 972h- | ATCC-24843 | h- | ATCC |
| IF170 | wildtype | CBS2777 |  | CBS |
| IF237 | wildtype | JB760 |  | Jürg Bähler (33) |
| IF247 | wildtype NCYC132 | ATCC-26192 |  | ATCC |
| IF37 | *ade6-210 leu1-32 ura4-D18 Δsir2::KanMX4* | SPBX120 | h+ | Blerta Xhemalce (79) |
| IF249 | *Δrqh1::KanMX4* | SPAC2G11.12 | h+ | Bioneer |
| IF234,IF235 | *Δsir2::KanMX4* | *sir2∆* | h- | this publication |
| IF256,IF258 | *Δrqh1::KanMX4* | *rqh1Δ* | h- | this publication |
| IF140 | *leu1-32* | TA23 | h- | Martin Kupiec (80) |
| IF230,IF231 | *leu1-32::LEU2-prNMT1-SIR2-GFH* | *Sir2*OE | h- | this publication |
| IF291 | *leu1-32 ura4-D18 gar2:mCherry:KanMX4* | Gar2 reporter | h- | Mitsuhiro Yanagida (81) |
| IF300 | *leu1-32 ura4-D18 gar2:mCherry:KanMX4, rqh1::Ura4MX6* | *rqh1Δ* + Gar2 reporter | h- | this publication |
| IF186 | *lys1, ura4, ade6-M210, his7::LacI-eGFP his7+, leu1::TetR-tdTomato Leu1+, Chr1 1.95Mb:LacO-NatMX6, Chr1 1.5Mb:TetO-HphMX* | CH2774,  Chr I reporter | h- | Christian Haering (72) |
| IF188 | *lys1, ade6-M210, his7::LacI-eGFP his7+, leu1::TetR-tdTomato leu1+, Chr1 2.49Mb:LacO-NatMX6, Chr2 3.6Mb:TetO-HygMX* | CH3245,  Chr II reporter | h- | Christian Haering (72) |
| IF396,IF397 | *leu1-32::pNMT1-SIR2-GF Leu1+, Δrqh1::KanMX4* | *rqh1Δ Sir2*OE | h- | this publication |

**Supplemental References**

1. Sveiczer A, Novak B, Mitchison JM. The size control of fission yeast revisited. J Cell Sci. 1996 Dec;109 ( Pt 12):2947–57. .

2. Wood E, Nurse P. Pom1 and cell size homeostasis in fission yeast. Cell Cycle. 2013 Oct 1;12(19):3417–25. DOI:10.4161/cc.26462.

3. Coelho M, Dereli A, Haese A, Kühn S, Malinovska L, DeSantis ME, Shorter J, Alberti S, Gross T, Tolić-Nørrelykke IM. Fission Yeast Does Not Age under Favorable Conditions, but Does So after Stress. Curr Biol. 2013 Sep;1–9. .

4. Jeffares DC, Rallis C, Rieux A, Speed D, Převorovský M, Mourier T, Marsellach FX, Iqbal Z, Lau W, Cheng TMK, Pracana R, Mülleder M, Lawson JLD, Chessel A, Bala S, Hellenthal G, O’Fallon B, Keane T, Simpson JT, Bischof L, Tomiczek B, Bitton DA, Sideri T, Codlin S, Hellberg JEEU, van Trigt L, Jeffery L, Li J-J, Atkinson S, Thodberg M, Febrer M, McLay K, Drou N, Brown W, Hayles J, Salas REC, Ralser M, Maniatis N, Balding DJ, Balloux F, Durbin R, Bähler J. The genomic and phenotypic diversity of Schizosaccharomyces pombe. Nat Genet. 2015 Mar;47(3):235–41. DOI:10.1038/ng.3215.

5. Xhemalce B, Kouzarides T. A chromodomain switch mediated by histone H3 Lys 4 acetylation regulates heterochromatin assembly. Genes Dev. 2010 Apr 1;24(7):647–52. DOI:10.1101/gad.1881710.

6. Weisman R, Roitburg I, Nahari T, Kupiec M. Regulation of Leucine Uptake by tor1+ in Schizosaccharomyces pombe Is Sensitive to Rapamycin. Genetics. 2005 Feb 1;169(2):539–50. DOI:10.1534/genetics.104.034983.

7. Nakazawa N, Nakamura T, Kokubu A, Ebe M, Nagao K, Yanagida M. Dissection of the essential steps for condensin accumulation at kinetochores and rDNAs during fission yeast mitosis. J Cell Biol. 2008 Mar 24;180(6):1115–31. DOI:10.1083/jcb.200708170.

8. Petrova B, Dehler S, Kruitwagen T, Hériché J-K, Miura K, Haering CH. Quantitative Analysis of Chromosome Condensation in Fission Yeast. Mol Cell Biol. 2013 Mar 1;33(5):984–98. DOI:10.1128/MCB.01400-12.
